# A Single Mechanism Can Account for Human Perception of Depth in Mixed Correlation Random Dot Stereograms

**DOI:** 10.1371/journal.pcbi.1004906

**Published:** 2016-05-19

**Authors:** Sid Henriksen, Bruce G. Cumming, Jenny C. A. Read

**Affiliations:** 1 Laboratory of Sensorimotor Research, National Eye Institute, National Institutes of Health, Bethesda, Maryland, United States of America; 2 Institute of Neuroscience, Newcastle University, Newcastle upon Tyne, United Kingdom; Justus-Leibig University, GERMANY

## Abstract

In order to extract retinal disparity from a visual scene, the brain must match corresponding points in the left and right retinae. This computationally demanding task is known as the stereo correspondence problem. The initial stage of the solution to the correspondence problem is generally thought to consist of a correlation-based computation. However, recent work by Doi et al suggests that human observers can see depth in a class of stimuli where the mean binocular correlation is 0 (half-matched random dot stereograms). Half-matched random dot stereograms are made up of an equal number of correlated and anticorrelated dots, and the binocular energy model—a well-known model of V1 binocular complex cells—fails to signal disparity here. This has led to the proposition that a second, match-based computation must be extracting disparity in these stimuli. Here we show that a straightforward modification to the binocular energy model—adding a point output nonlinearity—is by itself sufficient to produce cells that are disparity-tuned to half-matched random dot stereograms. We then show that a simple decision model using this single mechanism can reproduce psychometric functions generated by human observers, including reduced performance to large disparities and rapidly updating dot patterns. The model makes predictions about how performance should change with dot size in half-matched stereograms and temporal alternation in correlation, which we test in human observers. We conclude that a single correlation-based computation, based directly on already-known properties of V1 neurons, can account for the literature on mixed correlation random dot stereograms.

## Author Summary

Relating neural activity to perception is one of the most challenging tasks in neuroscience. Stereopsis—the ability of many animals to see in stereoscopic 3D—is a particularly tractable problem because the computational and geometric challenges faced by the brain are very well understood. In essence, the brain has to work out which elements in the left eye’s image correspond to which in the right image. This process is believed to begin in primary visual cortex (V1). It has long been believed that neurons in V1 achieve this by computing the correlation between small patches of each eye’s image. However, recent psychophysical experiments have reported depth perception in stimuli for which this correlation is zero, suggesting that another mechanism might be responsible for matching the left and right images in this case. In this article, we show how a simple modification to model neurons that compute correlation can account for depth perception in these stimuli. Our model cells mimic the response properties of real cells in the primate brain, and importantly, we show that a perceptual decision model that uses these cells as its basic elements can capture the performance of human observers on a series of visual tasks. That is, our computer model of a brain area, based on experimental data about real neurons and using only a single type of depth computation, successfully explains and predicts human depth judgments in novel stimuli. This reconciles the properties of human depth perception with the properties of neurons in V1, bringing us closer to understanding how neuronal activity causes perception.

## Introduction

**Fig 1:**
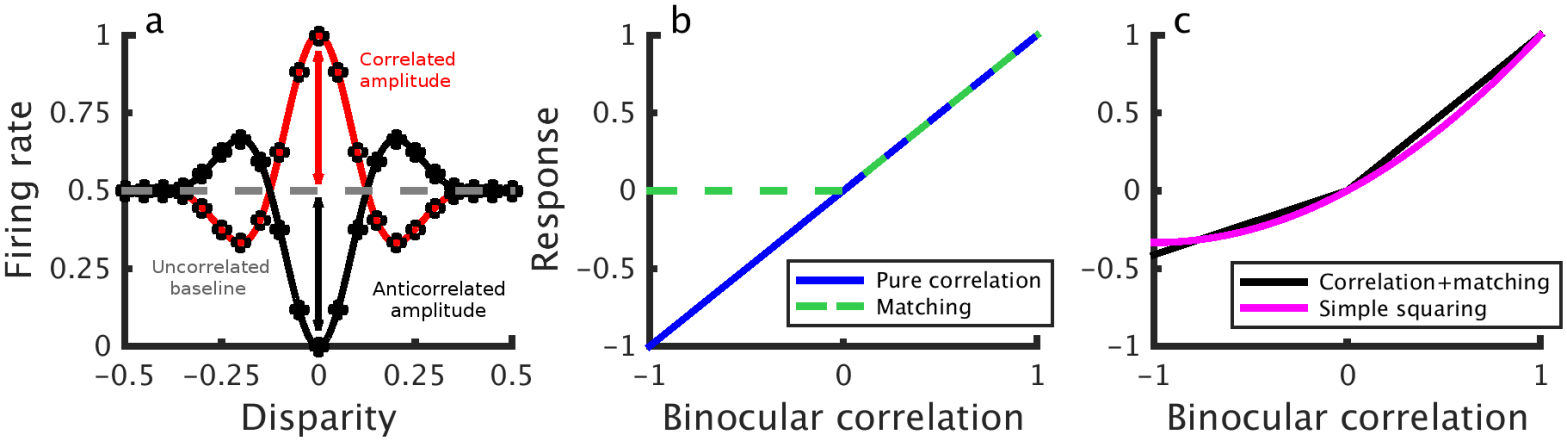
a) Response amplitudes in correlated and anticorrelated stereograms. Binocular energy model response to correlated (open circles, red line) and anticorrelated stimuli (solid circles, black line). The red arrow denotes the correlated amplitude, the black arrow the anticorrelated amplitude and the gray line the uncorrelated baseline response of the model cell. The amplitude ratio is the anticorrelated amplitude (black line) divided by the correlated amplitude (red line). b) The response at the preferred disparity as a function of binocular correlation (blue) which is linear, hence we call this a “pure” correlation computation. The green line shows this response after rectifying in the correlation domain—proposed as a separate computation by Doi & Fujita [16]. c) shows a linear combination of the two lines in b)—a single computation passed through a different nonlinearity (black). As shown, the two computations in b) and the linear combination of the two in c) are indistinguishable descriptions—b) contains two mechanisms, but their combined response is identical to c). If the two components in b) differ in some other respects (spatial or temporal properties) it may be possible to demonstrate that a single mechanism cannot reproduce the same behavior. No study has yet attempted to describe behavioral data with a single mechanism like c). We explore a mechanism based on a slightly simpler nonlinearity—squaring the response of the model in a (magenta line in c).

Stereoscopic vision is possible because the left and right eyes receive slightly different images of the world. This geometric arrangement gives rise to retinal disparity which can be used to extract depth information from a visual scene. A key challenge in stereo vision is determining which elements in the left eye’s image correspond to those in the right eye’s image. This computationally demanding task is known as the stereo correspondence problem and has been extensively studied [1–5]. The initial step in the solution of the correspondence problem is generally thought to be something close to a cross-correlation of the image patches in the left and right eye [6–8]. This view arose from work by Ohzawa, DeAngelis and Freeman (1990) [9] who proposed the very successful binocular energy model (BEM) for disparity selective V1 complex cells. The BEM provides a physiological instantiation of a correlation-type computation which accounts for the ability of these neurons to signal disparity in random dot stereograms (RDSs). Ohzawa et al [9], recording from V1 in the cat, used line stereograms and showed that inverting the contrast in one eye (i.e. making the line stereogram anticorrelated) also inverts the profile of the disparity tuning curve. Similarly, Cumming and Parker [10] inverted the binocular correlation of random dot stereograms and demonstrated that the profile of the disparity tuning curves of macaque V1 neurons also invert. The original binocular energy model is an example of a pure correlation computation as it predicts that the amplitude of disparity-related modulation should be equal for correlated and anticorrelated patterns. In other words, the amplitude ratio between the correlated and anticorrelated responses should be equal to 1. This is illustrated in Fig 1a, where the amplitude ratio is given as the ratio between the length of the black and red lines. However, many V1 neurons have amplitude ratios less than 1; the anticorrelated response is attenuated relative to the correlated response [10]. One explanation could be that an initial correlation computation is followed by a nonlinearity which results in responses that are not a linear function of correlation [11–13].

**Fig 2:**
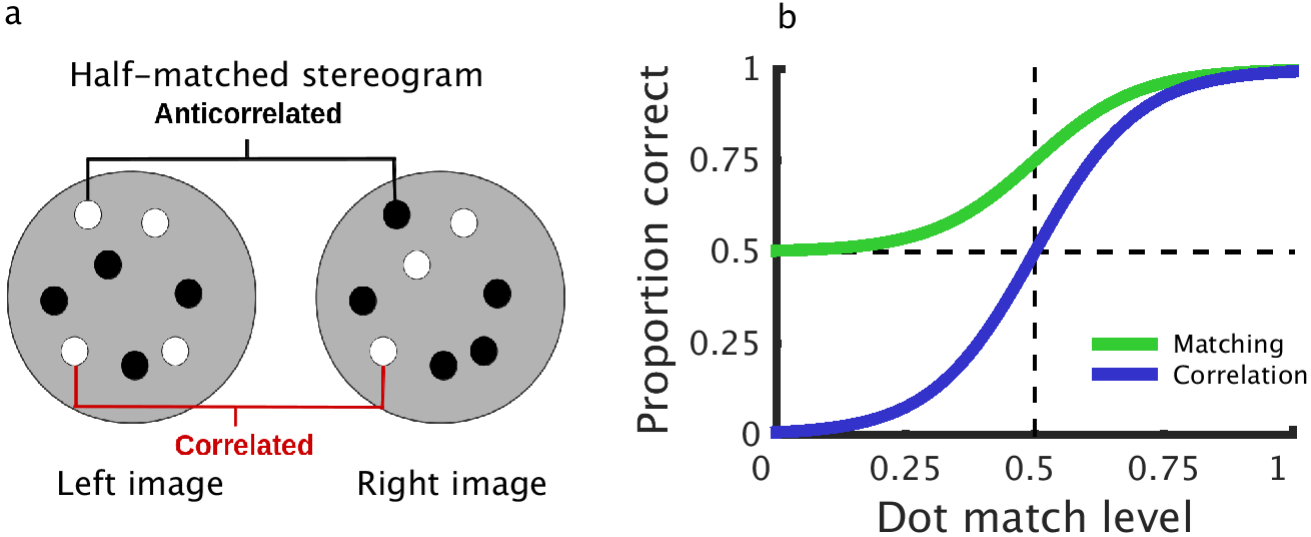
Correlation and matching computations in random dot stereograms. a) A schematic representation of a half-matched random dot stereogram. Half the dots are correlated, half the dots are anticorrelated, resulting in a binocular correlation of 0. b) Psychometric functions reflecting the operation of hypothetical matching and pure correlation computations (recreated from Doi et al [14]) as a function of dot match level. The pure correlation computation (blue line) signals reversed depth at a dot match level of 0 (fully anticorrelated), is at chance at a dot match level of 0.5 (half-matched) and performs perfectly at a dot match level of 1. The matching computation (green line) is at chance for anticorrelated stimuli and gradually increases to perfect performance with increasing percentage of matched dots. The horizontal line shows chance performance, and the vertical line marks a dot match level of 0.5, e.g. the half-matched stereogram shown in (a).

In a recent series of papers, Doi et al [14–16] have proposed that two distinct computations contribute to depth perception in cyclopean stimuli. They postulate a pure correlation mechanism, which depends linearly on interocular correlation, plus an additional “matching” computation, which in their most recent work [16] they have suggested may simply be the correlation mechanism plus an additional output nonlinearity. Under some circumstances, this is identical to a single mechanism with a nonlinear response to correlation (see Fig 1b and 1c). However, Doi et al [14,15] propose that the two mechanisms have distinct spatiotemporal integration properties, and so may be differentially activated by different stimuli. These conclusions are motivated by a series of ingenious psychophysical experiments in which the authors mixed correlated and anticorrelated dots within a single random dot stereogram (RDS). When half the dots in an RDS are correlated and half are anticorrelated (half-matched RDSs; Fig 2a), the global binocular correlation of the stimulus is 0. In this case, the authors argue, a pure correlation computation should not be able to detect depth. However, humans can perceive depth in such stimuli [14,15]. Doi et al [14,15] argue that this cannot be explained by a pure correlation mechanism, and propose an additional matching mechanism to account for these data. In addition to depth perception in half-matched RDSs, two pieces of evidence suggest that two separate mechanisms extract disparity in random dot stereograms. Doi et al [14] have reported that larger disparities tend to lead to decreased performance for half-matched RDSs and more reversed depth responses to anticorrelated RDSs. In a subsequent publication, Doi et al [15] reported a similar phenomenon in the temporal domain. They investigated dynamic random-dot patterns, in which the dot pattern is periodically replaced with a new random pattern with the same disparity and correlation. They showed that faster dot pattern refresh rates lead to poorer half-matched judgments and more reversed depth responses to anticorrelation. The authors argue that these results again reflect the weighted contribution of two separate mechanisms: one slow matching computation, responsible for fine disparity discrimination, and one rapid correlation computation, responsible for coarse disparity discrimination. Fig 2b illustrates schematically the performance they expect from these two computations in isolation.

While it is true that half-matched RDSs—stereograms with equal numbers of correlated and anticorrelated dots—have a mean binocular correlation of 0, it is possible that local fluctuations in correlation could be exploited to determine the stimulus disparity [15,16]. Doi et al [14,15] propose that these fluctuations are used by the matching computation, possibly in extrastriate cortex. However, the attenuated responses of V1 neurons to anticorrelated dots [10] makes it possible that even V1 neurons could encode disparity in these stimuli. V1 neurons respond more strongly to positive than negative binocular correlation [10]. If this attenuated response was generated by a simple output nonlinearity, then their responses to stimuli with high correlation variability may be greater than predicted from the mean correlation alone. In other words, the mean response of the cell may depend on the local correlation variability as well as the mean correlation. Doi & Fujita [16] explore this with a modified version of a cross-correlation computation which they refer to as “cross-matching”. Cross-matching computes the correlation between left and right images, but then follows this by half-wave rectification. Doi & Fujita [16] conclude that cross-matching has the necessary properties to serve as the computation underlying their putative match-based computations. Importantly, they still postulate that human stereo vision is subserved by separate pure correlation and match-based computations, with different spatiotemporal properties, and whose contribution to perception varies with properties of the stimulus. The proposition that these two mechanisms have different spatiotemporal properties is crucial if the two-mechanism model is to be distinguished from a single mechanism intermediate between the pure correlation and pure matching models. While differences in spatiotemporal properties are essential in order to separate “pure correlation” and “cross-matching”, changes in psychophysical performance with changes in spatiotemporal properties of the stimulus do not necessarily imply that there must be two mechanisms. In principle, changes in performance could be due to changes in the stimulus, even with a single-mechanism model. However, no one has yet explored whether a single-mechanism model can account for the results of Doi et al [14,15].

Here we explore the possibility that a single computation can explain depth perception in correlated, anticorrelated, and half-matched random-dot stereograms. Like Doi & Fujita [16], we use a model that can describe the attenuation observed in V1 neurons—a binocular energy model with an additional output nonlinearity—and show that this can explain responses to half-matched stereograms. Our scheme differs from theirs in that it does not suppose two distinct computations operating in cortex, but rather uses a single mechanism to explain all the psychophysical data. To explore this model, we first investigate the model responses to half-matched RDSs. We then show that this model can account for a range of previously documented psychophysical phenomena, including effects which Doi et al [14–16] have suggested are diagnostic of either a pure correlation or a match-based computation. Finally, we confirm two new predictions made by the model in human observers: that psychophysical performance should become worse with 1) decreased dot size in half-matched stereograms and 2) in response to rapid temporal modulation in correlation.

## Results

### Binocular energy model disparity tuning to half-matched stimuli

**Fig 3:**
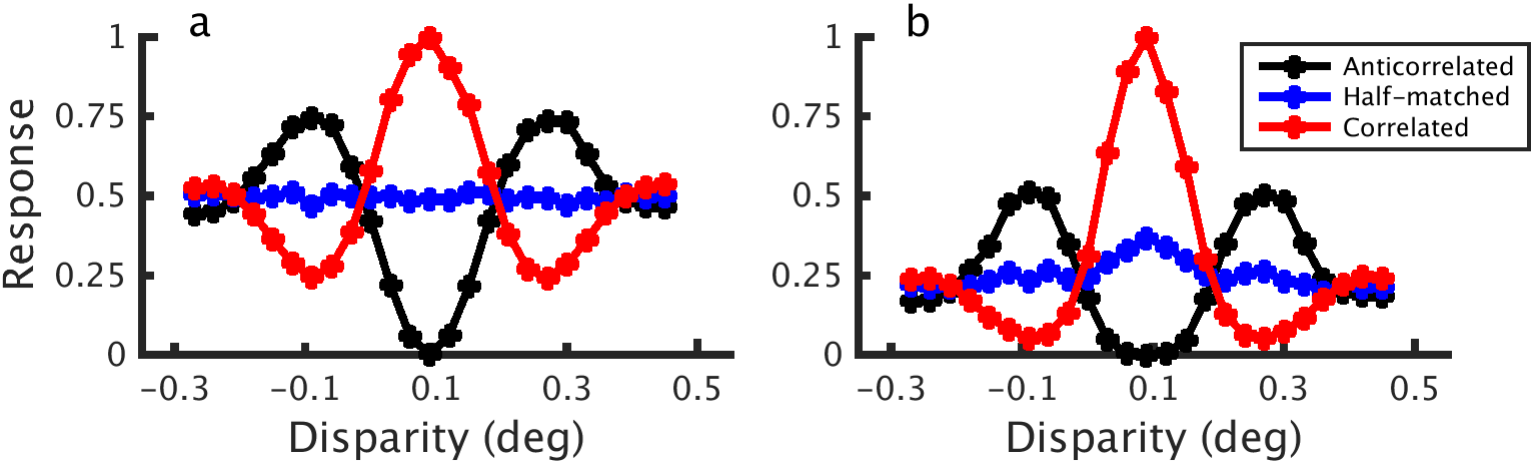
Binocular energy model tuning curves. Tuning curves from a binocular energy model in response to correlated, half-matched and anticorrelated RDSs for a binocularly linear model (a) and a model with an output nonlinearity (b).

We created binocular energy model (BEM) units by combining model binocular simple cells whose monocular receptive fields were in quadrature phase. This gives a model complex cell response *C* which is invariant to stimulus phase. As discussed above, the response of a BEM unit like this is a linear function of binocular correlation. We made the model nonlinear with respect to correlation by adding a static squaring output nonlinearity, giving a final response *C*^2^ (see Materials and Methods). The same model was also explored by Read, Parker, and Cumming (2002) [13]. We computed the response of both models to correlated, half-matched and anticorrelated random dot stereograms (RDSs) of various disparities. Disparity tuning curves for the BEM with and without an output nonlinearity are shown in Fig 3. For the binocularly linear BEM (Fig 3a), the model’s responses to correlated and anticorrelated stimuli have a characteristic symmetry about a horizontal line (the response to zero binocular correlation), i.e. the amplitude ratio between the correlated and anticorrelated responses is 1. For half-matched stimuli, the model has no disparity selectivity. When the model has a static output nonlinearity (Fig 3b), the correlated and anticorrelated tuning curves become asymmetric. This asymmetry leads to a modest, but very clear disparity tuning for half-matched RDSs with a peak at the neuron’s preferred disparity.

**Fig 4:**
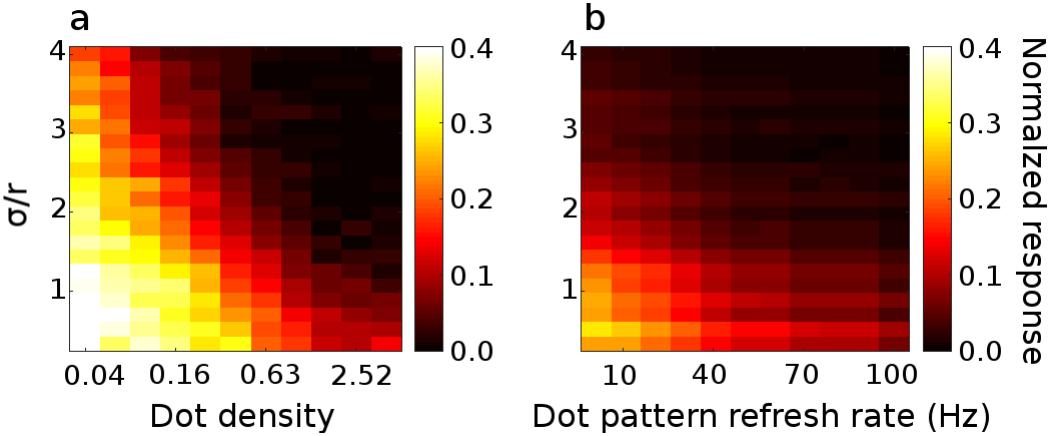
Effect of dot density, dot pattern refresh rate and relative receptive field size on disparity tuning in a model cell. a) Normalized half-matched response (Eq 1) as a function of density and relative receptive field size (density is here defined as the equivalent proportion of space the dots would occupy if they did not occlude one another). A relative receptive field size of 2 means that σ/r = 2, i.e. that the standard deviation of the monocular Gabor is twice the dot radius. Colors show the magnitude of the normalized response. A normalized response of 1 would indicate that the response to half-matched stimuli at the preferred disparity is equal to the correlated response; a normalized response of 0 means that the half-matched response is equal to the uncorrelated response. Decreasing the relative receptive field size and reducing the dot density both increase the variability in the correlation level and thus increase the half-matched response. The normalized responses were obtained by computing the average half-matched, correlated, and uncorrelated responses from 20 000 RDS per density-relative RF size combination. b) Normalized half-matched response as a function of dot pattern refresh rate and relative receptive field size. Decreasing the relative receptive field size again increases the half-matched response. Similarly, increasing the dot pattern refresh rate decreases the variability in binocular correlation and thus decreases the half-matched disparity tuning. The normalized responses were obtained by averaging across 5000 trials, 10 seconds in duration, for each frequency-relative RF size combination.

In the linear BEM (Fig 3a), the half-matched nature of the stimulus manifests itself as a variable firing rate, but not as a mean change [15]. The disparity tuning in Fig 3b arises because the expected value of a squared random variable depends on its variance: *E*[*X*^2^] = (*E*[*X*])^2^ + Var[*X*] (other choices for the exponent, as well as other nonlinearities, such as thresholding, will also yield a dependence on variance). For the model with an output nonlinearity, therefore, the high correlation variability in half-matched RDSs gets converted into a mean change in the firing rate. Because the tuning in Fig 3b is the consequence of fluctuations in local correlation over the RF, stimulus parameters that affect the variability of these local measures also affect the disparity tuning. Increasing the dot density decreases the correlation variability as there are more dots within a neuron’s spatial receptive field. Following earlier studies [14,15], dot density is defined as the proportion of the stimulus area that would be covered by dots if the dots were not allowed to occlude (although dots were allowed to occlude), hence the units are in proportion coverage. Having fewer, larger dots (while maintaining constant density) generally increases the correlation variability as a single dot fills a larger fraction of the RF with pixels sharing the same correlation. With more, smaller dots, the cell is integrating across a greater number of samples (since the cell is likely to see more independent dots within its receptive field) and so the variability is reduced. Because increasing the dot size while holding density constant is the equivalent of reducing the receptive field size in the model, we use the relative receptive field size, defined as σ/r, where σ is the standard deviation of the monocular Gabors, and r is the dot radius. The effect of density and relative receptive field size on disparity tuning in our model can be seen in Fig 4a, where the strength of disparity tuning for half-matched stimuli is plotted as a proportion of the modulation produced by correlated patterns with the same spatial parameters. We define this normalized mean response from the responses to the preferred disparity:

$$R_{norm}=\frac{\langle C_{hm}^{2}\rangle -\langle C_{uncorr}^{2}\rangle }{\langle C_{corr}^{2}\rangle -\langle C_{uncorr}^{2}\rangle }.$$ (1)

where $\langle C_{uncorr}^{2}\rangle$,$\langle C_{hm}^{2}\rangle$, and $\langle C_{corr}^{2}\rangle$ recepectively the mean response to uncorrelated, half-matched and correlated stimuli at the neuron's preferred disparity.

As the relative receptive field size decreases (i.e. smaller receptive field relative to the dot size), modified BEM cells signal disparity more vigorously to half-matched stimuli (relative to equivalent correlated stimuli). It is worth noting that while the amplitude ratio is large for very low densities, this does not necessarily translate to better psychophysical performance on the task. This is because at low densities, the responses to both correlated and anticorrelated patterns are much weaker, so the signal-to-noise ratio is lower. (The variations caused by the monocular image content dominate the model’s response). The effect of this is that there may be poorer performance at very low densities than at higher densities, despite the amplitude ratio being higher at low densities. Consider the case where the density is so low that on some trials no dots are presented in the RF. For these trials performance based solely on this neuron will be at chance, despite the high normalized response ratio averaged across many trials. Even performance based on many neurons would presumably suffer from a lower signal to noise ratio. We will explore the effects of signal and noise in simulations of a psychophysical task below.

Just as increasing the spatial extent of the receptive field (relative to dot size) reduces variability in binocular correlation seen by the model, so increasing the temporal extent of the RF (relative to the pattern update frequency) reduces variability. We explore this using a fixed RF and a changing dot pattern refresh rate. As refresh rates increase, the model cell is integrating more dots within its temporal window, reducing fluctuations in the binocular correlation. Modified BEM cell responses are shown in Fig 4b for different receptive field sizes and pattern refresh rates. For low refresh rates and small RF sizes, the cell exhibits substantial disparity tuning to half-matched stimuli. As in Fig 4a, when the RF size increases relative to the dot size, the disparity tuning to half-matched stimuli decreases since the cell is integrating across more dots. Similarly, as the dot pattern refresh rate increases, the half-matched disparity tuning decreases since the cell is again integrating across more dots (but now across time rather than across space).

These simulations demonstrate two key properties: the model exhibits less disparity tuning to half-matched stimuli as 1) the receptive field increases in size relative to the dot size, and 2) the refresh rate increases relative to the temporal integration period of the neuron. The first finding is noteworthy because it was observed in Doi et al [14] that human observers are better at reporting depth in fine disparity half-matched RDSs than in coarse disparity ones. Given the effects of RF size we show, this observation might be accounted for by the well-known size-disparity correlation [7,17–20], since coarse disparity detectors tend to have larger receptive fields than fine disparity detectors. The second finding is noteworthy because Doi et al [15] reported that performance to half-matched stimuli also decreased with increasing pattern refresh rate. The authors interpreted this as a shift from a matching computation at low refresh rates to a pure correlation computation at high refresh rates. In the current framework, the decreased disparity tuning with refresh rate reflects temporal integration within a single correlation-based computation, rather than differential activation of two distinct computations with different spatiotemporal properties. Although the match-based computation hypothesized by Doi et al [15,16] is similar to our modified correlation-based computation, in our framework, only a single computation is involved. Indeed, our model neurons could be described as the sum of a matching computation and pure correlation computation, just as illustrated in Fig 1c, but this is achieved by a single mechanism. Unless the two components differ in some other way (e.g. temporal response), the two descriptions are identical.

### Psychophysical decision model

Clearly, the fact that our version of the BEM can signal disparity in half-matched stereograms makes it possible that this explains human psychophysical performance. Additionally, our model neurons lose disparity tuning to half-matched stimuli with increasing receptive field size and dot pattern refresh rate, which is also in agreement with the psychophysical literature [14–16]. Finally, the model neurons show weaker responses to anticorrelated dots, which produce weak or absent depth sensations [14,15,21–23]. Thus, at least qualitatively, the signal strength in these model neurons parallel all of the psychophysical phenomena that have been used to suggest two stereo mechanisms. However, these manipulations also influence the ratio of the signal to noise, so the response amplitudes described above cannot simply be compared to psychophysical performance. To test more formally whether our model can explain these psychophysical phenomena, we simulated responses of a small population of neurons and made the perceptual decision based on a straightforward linear readout of the population activity.

**Fig 5:**
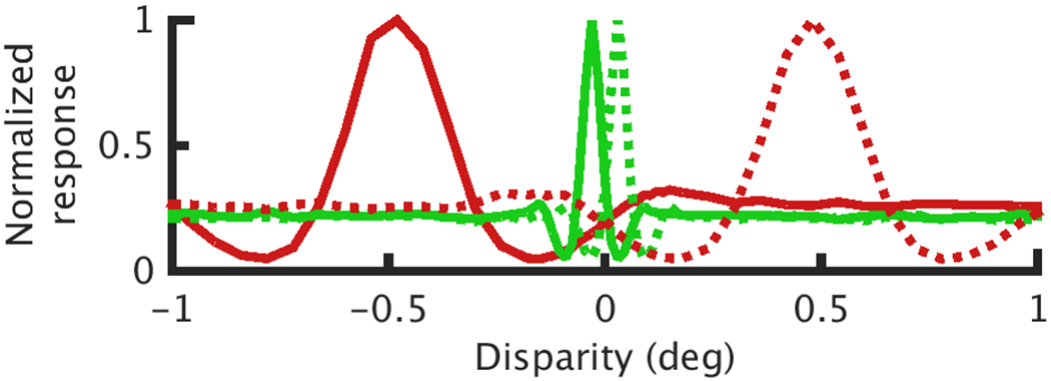
Disparity tuning curves for binocular energy model units with a squaring output nonlinearity in response to 100% correlated RDSs. Green lines show fine disparity tuning curves, red lines show coarse disparity tuning curves. Dashed lines indicate cells tuned to positive disparities, while solid lines indicate cells tuned to negative disparities. The tuning curves were constructed by computing the responses of four model cells to correlated RDSs with disparities in the range [−1°, 1°]. 5000 RDSs were used per disparity.

The population and decision rule are described in detail in the Materials and Methods section. Briefly, our starting-point was the modified binocular energy model units discussed above, whose response is denoted by *C*^2^ in Eq 1. For neurons with different RF sizes, we scaled these responses, such that the cells had equal mean responses to correlated stimuli at their preferred disparity. The disparity tuning curves of these cells are shown in Fig 5 in response to correlated RDSs. The peak of the disparity tuning curves are identical for all cells, but the uncorrelated baseline is slightly lower for the fine disparity neurons (see Methods). We then applied Gaussian noise, independently for each unit, with variance proportional to the response at any time and the constant of proportionality being a free parameter. When using a fixed stimulus duration, higher pattern refresh rates mean that model responses average over a larger number of RDS images, reducing the stimulus-driven variability between trials. We therefore fit the noise parameter separately for each frequency. If an equal number of frames is used (and hence varying stimulus duration), the same results can be reproduced with fixed noise across frequencies. For simplicity, we restricted ourselves to four preferred disparities (−0.48°, −0.03°, 0.03°, 0.48°), and assumed that receptive field size scaled with disparity magnitude according to σ = 0.023 + 0.41|Δx|. In simulations using only one cell per disparity, random fluctuations in the monocular image content led to performance that was poorer than human subjects. We therefore included multiple cells for each disparity, differing only in their locations on the retina. Each cell had non-overlapping receptive fields that were otherwise identical. We found a good match to human performance using 40 cells per disparity, for a total of 160 cells in the model population.

For the decision rule, we first created opponent cells by taking the difference of a squared energy model neuron and its “antineuron”, i.e. the neuron at the same location in the retina but with preferred disparity differing in sign. To make the decision, each opponent cell’s response was summed across time points to obtain *R*^(*i*)^ the overall opponent (squared) energy for the *i*th neuron on a given trial (Eq 7). A negative value for any given neuron-antineuron pair means that the pair signals a negative (near) disparity, while a positive value means that the pair signals positive (far) disparity. To obtain a decision, we summed the activity across the pool of neuron-antineuron pairs. If this summed value was negative, the model reported that the stimulus had a negative disparity, and vice versa.

**Fig 6:**
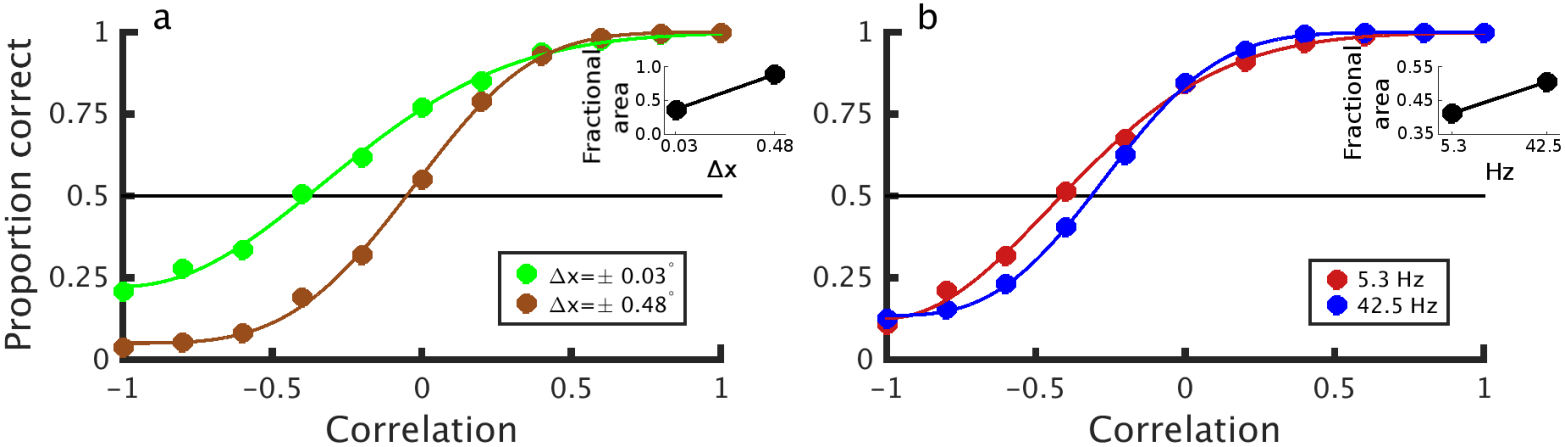
Simulated psychophysical performance. Performance of the model (proportion correct responses) as a function of binocular correlation. A binocular correlation of 0 here indicates half-matched stereograms, i.e. stimuli with an equal number of correlated and anticorrelated dots. a) Model performance to fine disparity stimuli (±0.03°, green line) is better than in response to coarse disparity stimuli (±0.48°, brown line) for many correlation values. b) Model performance to stimuli with low pattern refresh rate (5.3Hz, red line) is better than to stimuli with high refresh rate (42.5Hz, blue line). In a) all stimuli were updated at 21.25Hz, and in b), all stimuli were presented at a disparity of ±0.03°. Noise was fitted to match the performance of human observers. The insets in both figures show the fractional area, which quantifies the odd-symmetry component of the fitted psychometric function. A fractional area of 1 means that the psychometric function is completely symmetric (deviations from chance performance are exactly proportionate for anticorrelated and correlated stimuli), whereas a fractional area of 0 means that all of different-from-chance performance occurs at positive correlation values (i.e. the psychometric function is completely odd-symmetric).

We first consider the effect of disparity magnitude on stereo depth perception in half-matched RDSs. As Fig 4 shows, large dots (relative to RF size) produce stronger responses to half-matched stereograms than small dots. For a stimulus with a fixed dot size, this means that neurons with smaller RFs give stronger disparity signals in half-matched stereograms. Because we include a size-disparity correlation in our model, fine disparities will elicit responses predominantly from neurons with smaller RFs. These cells will see larger correlation fluctuations because of their small RF size and thus have a larger response in the half-matched condition. The results from the simulations are shown in Fig 6a. For half-matched stimuli (correlation of 0) the model performs better in response to fine disparity stimuli than to coarse disparity stimuli. This remains true across a range of correlation values, because even for low correlation values there are local fluctuations in the correlation level. The fine disparity model cells are more sensitive to these fluctuations than the coarse disparity cells, which leads to a leftward shift in the psychometric function. The upward shift at fine disparities arises because here all cells contribute to the disparity judgment (often with opposing signals, see Fig 5). However, for coarse disparities, the coarse cells dominate the decision to anticorrelated stereograms. These shifts are similar to that observed by Doi et al [14], but our account invokes only a single mechanism.

Next we consider the effect of dot pattern refresh rate on model performance. We used the same model as for the disparity magnitude simulations, but used fine disparity stimuli. We presented RDSs at two different frequencies: 5.3Hz and 42.5Hz, with a stimulus duration of 1.5s. Fig 6b shows the psychometric functions for the same model in response to low and high refresh rates for fine disparities (0.03°). As the pattern refresh rate increases, the psychometric function moves rightward. This shift occurs because the neuron is integrating across more dot patterns (in this case over time rather than space) which reduces local fluctuations in correlation. A similar result was obtained using an equal number of stimulus frames rather than equal stimulus duration.

Our model produced weak reversed depth in response to anticorrelated stimuli. This is similar to some human studies [14,15,22], but the reversed depth reported by our model was also generally stronger than that reported in the literature. This partly reflects the fact that our model cells modulate their activity more than typical V1 neurons. A final output exponent greater than 2 would reduce this, but we present data for the simplest model as it is more tractable. Additionally, responses to anticorrelated stimuli are influenced by factors that are not readily incorporated into simple models. For example, when a zero-disparity annulus is also anticorrelated, depth perception is abolished [22,24], but when the surround is correlated, depth is sometimes reported [14,15,23]. No existing models provide an account of this effect of the surround. While a sufficiently complicated model could doubtless explain this important phenomenon, adding additional parameters to the model would make it harder to interpret the success in explaining the results we discuss here. To this extent, the description of reversed depth in our model (and all other extant models) is a simplification. In our model, the extent to which reversed depth is reported is quite sensitive to the shape of the disparity tuning curves used for model neurons. Despite this, the model does make clear that the information available about negative correlations is influenced by these stimulus manipulations. Our configuration illustrates that it is possible to explain the results of Doi et al [14,15], including reversed depth, using a single mechanism.

Here we have have used a linear readout of population activity, which is a parsimonious method of neural decoding, and has frequently been used before [25–27]. It is possible that a more sophisticated decision model could yield performance even more similar to the human observers. Indeed, more complex models, such as those incorporating Bayesian priors [28], or using maximum likelihood decoding [29], have been shown to capture human psychophysical performance across a range of stimuli and tasks. However, given the simplicity of our model population (only two cell types), and the fact that the model cells do not faithfully reflect the properties of V1 neurons (which show stronger attenuation to anticorrelated stimuli), exploring more complex decision rules seems inappropriate.

### The effect of dot size on human and model performance

Our model neurons show reduced responses to half-matched stimuli as dot size (relative to RF size) is reduced. If this correctly captures the nature of signals used to perceive depth, we should expect human performance also to depend on dot size to half-matched RDSs, but not for 100% correlated stereograms. We therefore examined the effect of dot size on depth perception.

**Fig 7:**
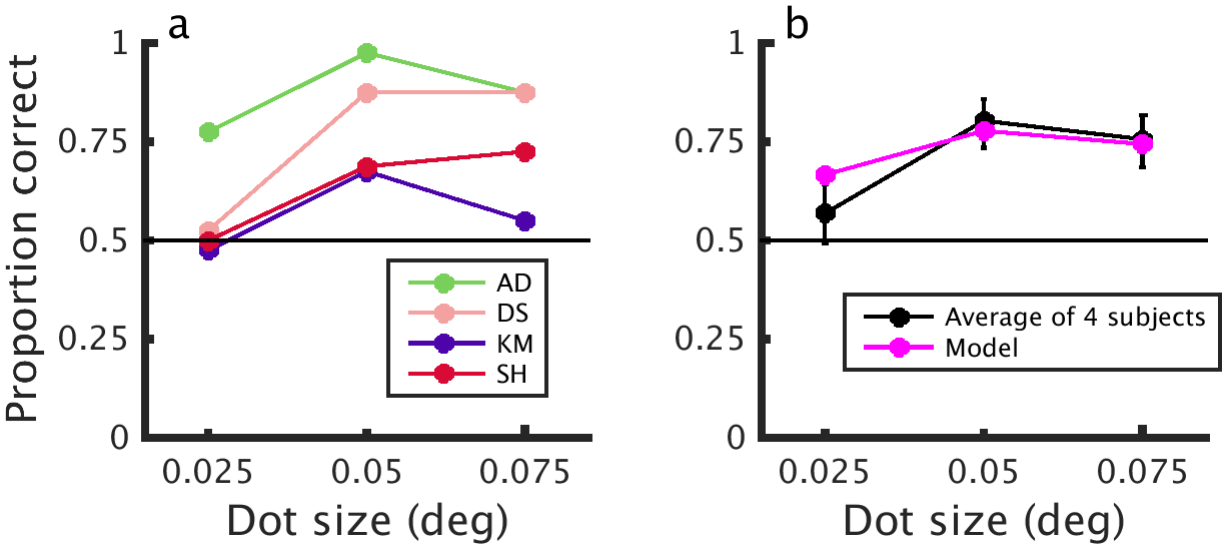
The effect of dot size in half-matched RDSs. a) Psychophysical performance for four human observers in response to dynamic half-matched RDSs of three different dot sizes presented at 21.25 Hz dot pattern refresh rate. Each data point shows a minimum of 40 trials for each dot size per subject. b) Average performance for the four human observers and for the model shown in Fig 5. Error bars show binomial 95% confidence intervals. The noise-level was fitted to match the human observers. Dot density for all stimuli was 24%.

Fig 7a shows the proportion correct as a function of dot size for 4 observers. For the smallest dot size (0.025°), performance is not significantly different from chance for three of the observers using 95% binomial confidence intervals. We used a Monte-Carlo simulation to test if dot size had a significant effect on the variance in performance and found that it did (*P* = 6.3 × 10^−5^). correlated stimuli, all observers performed at ≥ 95% correct for all dot sizes. Thus, the decreased performance in response to small dot half-matched RDSs cannot be attributed to changes in the spatial content of the monocular images. The decreased performance is consistent with predictions made by energy model units with a simple output nonlinearity. Fig 7b compares the average response of the human observers with the psychophysical decision model introduced earlier (Fig 6). Both the model and the average human performance show a similar decline for the smallest dot size.

### Alternating-correlation stereograms: Temporal half-matching

**Fig 8:**
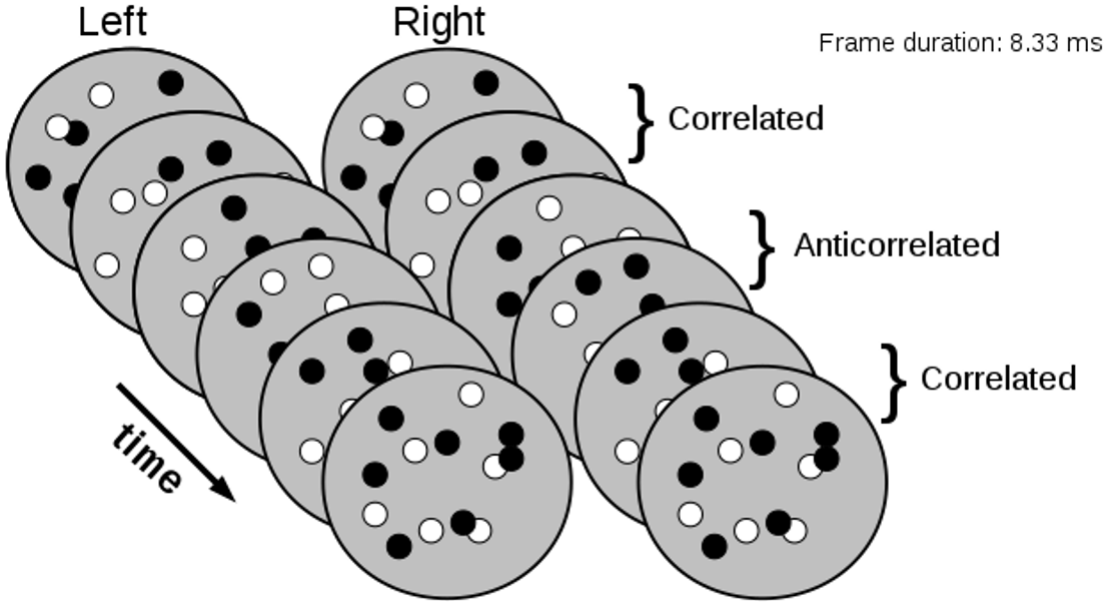
Alternating-correlation random dot stereograms. In the illustration, the observers first see two correlated frames, then two anticorrelated frames, then two correlated frames and so on for 500 ms. The dot pattern is updated at 120Hz with the correlation alternation rate varying across trials.

We showed above that the effects of pattern refresh rate on performance in half-matched stereograms can be explained by the effects of temporal integration on local fluctuations in correlation. Doi et al [15] propose a different explanation, which is that the matching process is slow and so the rapid changes in monocular patterns disrupt the matching computation, leaving the pure correlation computation to dominate at fast refresh rates. In order to provide an additional test of these hypotheses we introduce a new stimulus in which the monocular pattern refresh rate is always high, but binocular correlation changes at different rates. Each random dot pattern is either 100% correlated or 100% anticorrelated (with the same disparity), and the correlation value alternates (while monocularly every new monitor frame shows a new pattern, as illustrated in Fig 8). We then explored the effect of changes in frequency with which the correlation alternates. By presenting dot patterns at very rapid pattern refresh rates, we should be able to keep the contribution of any putative slow matching computation to a minimum, independent of alternation rate. Doi et al [15] showed that the energy seen by their sustained, matched-based mechanism fell by a factor of 2 as the pattern refresh rate increased from 5.3Hz to 43Hz [15]. At 43Hz, the highest refresh rate they could present, their sustained and transient channels were seeing equal stimulus energy. In our CRT mirror stereoscope, we use a pattern refresh rate of 120Hz. According to Doi et al’s [15] model, this will ensure that the transient channel feeding into the pure correlation mechanism is driven far more strongly than the sustained channel feeding into the match-based mechanism. Perception in this stimulus should therefore be dominated by the pure correlation mechanism.

**Fig 9:**
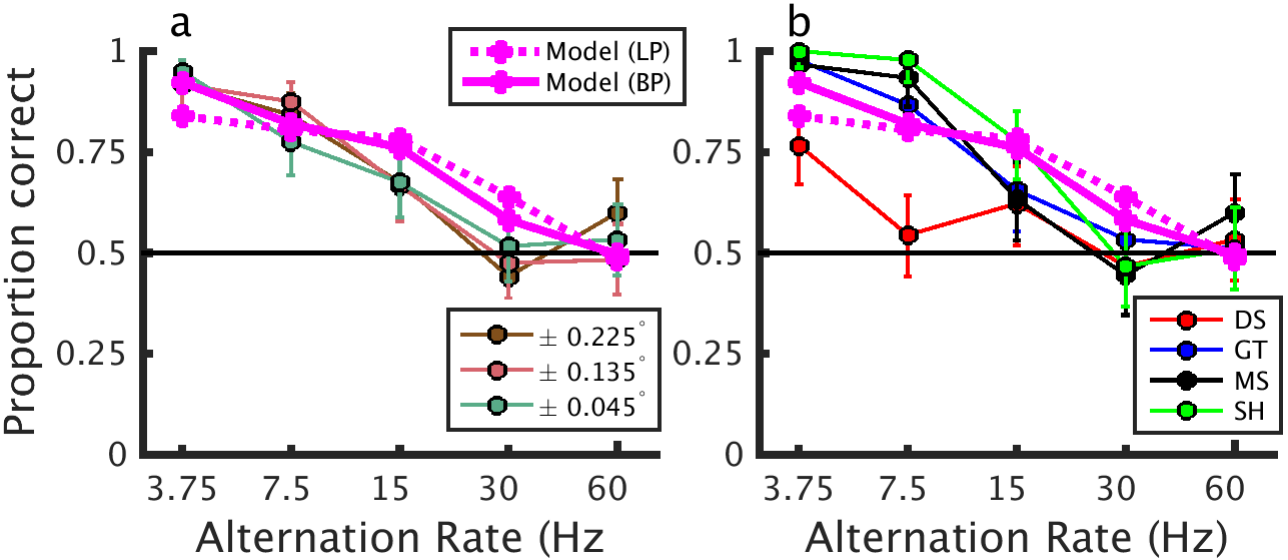
Psychophysical performance to alternating-correlation stereograms. Performance of human observers on a front-back discrimination task with alternating-correlation random dot stereograms. a) Each line shows the averaged performance of all subjects for each disparity magnitude. b) Each line shows the performance of an individual observer averaged across the three disparities. The solid magenta line in each plot shows model performance with our band-pass temporal kernel [Model (BP)], and the magenta dotted line shows the model performance with the low-pass temporal kernel used in Doi et al [15] [Model (LP)]. In both cases, the model was shown a disparity of ±0.03°, and noise was fitted to match human observers.

However, Doi et al’s [15] model also predicts that even though the pure correlation mechanism is strongly driven, it must perform at chance in this task. Their definition of a pure correlation mechanism is one that outputs 100% veridical depth for 100% correlated stimuli, 100% reversed depth for anti-correlated stimuli, and is at chance (50%) for half-matched stimuli. Now, at alternation rates which are slow compared to the temporal kernel of this mechanism, the observers are simply seeing a rapidly updating stimulus which periodically flips between being correlated and anticorrelated. Let’s say this stimulus has a near disparity. Doi et al’s [15] pure correlation mechanism will report alternately “near” and “far” as the correlation flips. Since we randomised the phase of our alternation, over many trials there is no way for their pure correlation mechanism to report “near” more or less often than “far”. On average, therefore, performance must be at chance. The situation is no better for alternation rates which are fast compared to this mechanism. There, both correlated and anticorrelated frames fall within the temporal integration window. The stimulus is effectively half-matched, and by definition, Doi et al’s [15] pure correlation mechanism must be at chance. Thus, a pure correlation mechanism, as defined by Doi et al [15], cannot contribute to above-chance performance with this stimulus. Their match-based mechanism can contribute in principle, but their conclusions about its temporal properties—that it is temporally low-pass—makes depth discrimination in rapidly updating stimuli, such as the alternating-correlation stereograms, a particularly demanding task. According to Doi et al [15], depth perception makes use of “a simple, correlation-based representation for more dynamic (faster) and coarser features, and a complex, match-based representation for less dynamic (slower or stationary) and finer features” [15]. The implication is that for very rapidly updating stimuli, the relative contribution of the matching computation to depth perception should be very small. Thus, this task presents a particular challenge because the near/far judgment must be based on weak signals from the slow matching computation, and stronger, but alternating and conflicting signals from the fast correlation computation. Yet as Fig 9 indicates, our human observers performed well above chance for alternation rates below 30Hz.

We compared the responses of human observers to alternating-correlation RDSs of various alternation frequencies with that of the psychophysical decision model used earlier. Fig 9a shows psychophysical performance in response to alternating-correlation RDSs averaged across four subjects for the three disparities employed. Clearly, stimulus disparity had virtually no effect on this task, where task difficulty was manipulated by increasing the alternation rate. Individual psychometric functions, averaged across disparities, are shown for each subject in Fig 9b. In both plots, model responses are shown in magenta. At alternation rates below 4Hz, the human observers make accurate judgments, but as the alternation rate increases, performance decreases. At intermediate alternation rates, the human observers can still do the task, but crucially, as the alternation rate increases beyond about 20Hz, the correlation variability decreases and the performance of human observers falls to chance. We conclude that our model accounts excellently for human performance in this stimulus. Using the temporal kernel defined by Doi et al [15] for the matching computation, we find a slightly less good fit to the data (see dotted line in Fig 9). The main difference is at slow alternation rates, where subjects perform very well as does our mechanism with a bandpass kernel. This model is unable to produce good performance here because of the high monocular pattern refresh rate and its lowpass kernel. Presumably, performance would be even worse in a two-mechanism model where the weak signal from the low-pass matching computation competes with a much stronger, alternating signal from the pure correlation computation.

## Discussion

The first step in stereoscopic depth perception is the extraction of disparity from the left and right images. The success of the binocular energy model in describing the responses of disparity-selective cells in primary visual cortex [9,10,13] led to correlation-based schemes becoming the dominant explanation for human stereo vision [8,30,31]. Disparity-selective cells in V1 show the hallmark of a correlation computation: inverting the binocular correlation of the stimulus inverts their response, whereas a unit looking for matching features would simply cease to detect any disparity after this manipulation. Recently, Doi et al [14–16] have postulated that two distinct computations extract disparity in dense random dot stereograms: one extracts disparity by computing the binocular correlation, while the other extracts disparity by computing the binocular match level of the image. Doi et al [14,15] cite three key observations as evidence for the existence of this match-based computation. First is the fact that humans correctly report depth in random dot stereograms made of equal numbers of correlated and anticorrelated dots [14,15]. Second, performance to these half-matched stimuli degrades with disparity magnitude, in a way that is not seen with correlated RDS [14]. Third, in dynamic stimuli where the dot pattern is regularly refreshed, performance declines with increasing refresh rate for half-matched, but not correlated, stereograms [15].

### A single model accounts for depth judgments in half-matched random-dot patterns

Here we show that all three of these observations can be explained by a model which uses a single nonlinear correlation-based mechanism (the BEM with a static output nonlinearity). The nonlinearity we propose is similar to the nonlinear “cross-matching” model proposed by Doi & Fujita [16]—our proposed mechanism is well described by the linear sum of a “pure-correlation” mechanism and a “cross-matching” mechanism (Fig 1c). Thus the two accounts are closely related. If the pure correlation filter and cross matching filter have the same monocular spatiotemporal RFs, the two accounts are indistinguishable. If the different computations are associated with filters that have different spatiotemporal properties it becomes possible to distinguish one mechanism from two, as two mechanisms will then predict different psychophysical performance. This is exactly what Doi et al [14,15] propose to explain psychophysical changes that occur with pattern refresh rate and disparity magnitude. We point out here that many of those changes in psychophysical performance might occur because of the effects of stimulus changes on the activity of a single mechanism. Doi et al [14–16] do not report any simulations with single-mechanism models, and so never test the null hypothesis that a single channel suffices. Our results suggest that it may. Importantly we do not claim to falsify a two-computation hypothesis—the isomorphism shown in Fig 1c means that any data described by a single computation can also be described with two. All three of these mechanisms (pure correlation, cross-matching, and the BEM with an output nonlinearity) could be described as “correlation-based”, since they all start by computing correlation.

Adding an output nonlinearity to the BEM allows the neurons to signal disparity in half-matched stereograms because the correlation fluctuations are converted to a mean firing rate through this nonlinearity. (When there is no nonlinearity, the correlation fluctuations manifest as a variable firing rate, but do not lead to a change in the mean). It follows from this that the larger the correlation fluctuations, the greater the disparity tuning to half-matched stimuli. We showed that the effect of disparity magnitude on half-matched depth perception can be explained if larger receptive fields are used to detect larger disparities (the well-known size-disparity correlation [7,17,19,20]). A similar point was noted by Doi et al [15], who found that larger receptive fields decrease the response variability of standard energy model cells, and by Doi & Fujita [16] who extended these findings to a “cross-matching” computation. Along the same lines, the observation that depth perception is compromised in rapidly changing half-matched RDSs is compatible with temporal integration within the correlation mechanism, and does not imply a qualitative shift to a different computation.

This model correctly predicts our finding that psychophysical performance decreases with smaller dot size, and states that this is because smaller dots tend to decrease the local correlation variability. It also correctly predicts that alternating the correlation over time should decrease psychophysical performance because of a reduction in the effective variability in binocular correlation. This is particularly interesting since the alternating stimulus presents particular challenges for both of the mechanisms proposed by Doi et al [15]. Their pure correlation mechanism should be at chance since, by definition, it reports opposite depth sign for opposite correlations, and thus reports either depth sign with equal probability for our alternating stimulus. Our single-mechanism model can straightforwardly account for depth perception in these stimuli. Our model has a single, fixed temporal kernel, and yet it can account simultaneously for the effects both of pattern refresh rate (Fig 6b) *and* of alternation rate (Fig 9). Indeed our model describes the data somewhat better than the “cross-matching” mechanism, most tellingly at low alternation rates. Here subjects are close to 100% correct, whereas the sustained temporal properties of Doi et al’s [16] cross-matching mechanism means that it only reaches 80% correct.

### Aspects of depth perception still unexplained

The differing contrast polarity of individual dots is a key feature that allows half-matched RDSs to be created. It may therefore be that the psychophysical performance with half-matched RDSs is related to an earlier observation, that human stereopsis performs better on random dot stereograms made up of mixed black and white dots than on stereograms made up of only one polarity [32,33]. No correlation-based model has yet provided an account of this phenomenon [33]. Like Doi et al [14–16], Harris & Parker (1995) [32] explained their result in terms of a mechanism which matches same-contrast dots (using independent “on” and “off” channels). However, it is not clear that independent on and off channels can explain the even greater benefit produced by mixed polarity dots at low correlation [33]. It may be that the inclusion of nonlinearities like those we use here, or the “cross-matching” of Doi & Fujita [16] would allow a single mechanism to explain the benefits of mixed-polarity dots, but this has yet to be demonstrated.

It has long been recognized that in some situations stereo correspondence that is not based on correlation can be exploited. For example, it is well-known that monocular occlusion (i.e. objects seen by one eye but occluded in the other) can contribute to the perception of depth in humans (so-called da Vinci stereopsis [34]). Additionally, patients with binocular vision disorders such as strabismus may show no depth perception with cyclopean stereograms, while having measurable stereoacuity in images with monocularly visible contours [35–37]. For isolated monocular targets, humans can correctly report depth for larger disparities than is possible in random dot patterns [38,39]. This suggests that the human visual system may also contain a separate algorithm, which enables a coarse form of stereopsis even when the correlation-based system is damaged [40], at least for sparse images consisting of a small number of monocularly visible objects. There is some evidence suggesting that this system can use head-centric rather than retinotopic coordinates [40–42], implying an extrastriate locus. Importantly, however, the match-based computation proposed by Doi et al [14–16] is quite different from this mechanism, as it would have to operate on dense random dot patterns.

Additionally, in human observers, depth perception in half-matched and anti-correlated stereograms requires the presence of an adjacent correlated region [14,15]. This may be related to humans’ greater sensitivity to the relative disparity between adjacent surfaces than to the absolute disparity of a surface in isolation. However, the presence of a correlated surround is not sufficient for reversed depth in anticorrelated RDSs [21,43]. As these observations indicate complex interactions between different regions of the visual field, it is inevitable that they cannot all be explained by models like ours (or that of Doi et al [16]) that consider only the responses of a population of neurons at one location. That a local model exploiting a single mechanism successfully explains so many phenomena strongly suggests that a single mechanism is responsible.

### Properties of V1 neurons

This mechanism closely resembles the known properties of disparity tuning in V1. The critical property is that V1 neurons show weaker tuning to anti-correlated than to correlated stimuli. The reduction becomes more pronounced in later areas [44,45], but is already present in V1 [10]. This asymmetry suggests that V1 neurons should, weakly, encode disparity in half-matched stimuli. This together with the tendency for V1 neurons tuned to large disparities to have larger receptive fields [46] can account for all the psychophysical data regarding half-matched stimuli. As we have shown, a model based on these ideas provides an excellent account of human performance from previous studies [14,15] (Fig 6) and also predicts performance on new stimulus manipulations (Figs 7 and 9). Importantly, we use one model while only fitting a noise parameter to explain the psychophysical results in Figs 6, 7 and 9. It is important to note, though, that no one has yet examined the response of V1 neurons to half-matched stereograms. Our model of V1 neurons captures their weaker tuning to anti-correlated stereograms, and predicts that this results in weak tuning to half-matched stimuli. Yet until this prediction has been directly tested in V1 neurons, we cannot be sure it occurs. If V1 neurons do not show disparity tuning for half-matched stimuli, this would give much greater credence to the idea of a separate dot-matching computation in extrastriate cortex. Our laboratory is currently exploring this question.

## Materials and Methods

All simulations were implemented in BEMtoolbox—a custom Matlab toolbox for simulating binocular neurons. The toolbox is available at http://github.com/sidh0/BEMtoolbox. All code used in the current manuscript is available online at http://github.com/sidh0/hcr16_ploscb (requires BEMtoolbox).

### Random dot stereograms

We created dynamic random-dot stereograms with a varying number of correlated and anticorrelated dots as described by [14,15]. Black and white circular anti-aliased dots, 0.09° in radius, were painted on a gray background. When the stimulus was half-matched, half the dots were correlated, i.e. had the same luminance in both eyes, while the other half were anticorrelated, i.e. drawn black in one eye and white in the other. There were on average equal numbers of black and white dots for each correlation value. Dots had zero binocular disparity except within the central 2.5° of the stimulus. The surrounding annulus had a width of 1°. The stimulus thus depicted a disparate disk either in front of or behind a zero-disparity background. No subpixel disparity was used. Unless otherwise specified, the dot density was 24%, meaning that if none of the dots overlapped, they would have occupied 24% of the stimulus area. The dots were, however, allowed to occlude one another. The dots were painted in random order so as to prevent any cues arising from occlusion due to either correlated dots systematically occluding anticorrelated dots or the surround dots systematically occluding the center dots. The above applies to both the human psychophysics and the simulations. For the psychophysical investigations, the background was kept 100% correlated for consistency with [14,15]. For all simulations, 292 x 292 pixels were used to simulate the stimulus such that 1 pixel corresponded to 0.03 degrees. When time was incorporated into the model, the simulations were carried out at a temporal resolution of 1 ms.

For the dot size experiment (Fig 7), we created half-matched (binocular correlation of 0, match level of 0.5) and correlated RDSs (binocular correlation of 1, match level of 1). We used three different dot sizes: 0.025°, 0.05°, and 0.075°. The dots were circular and anti-aliased. The surrounding annulus was always correlated as per Doi et al [14,15]. The stimuli were presented at a dot pattern refresh rate of 21.25 Hz, for 500 ms, and at a disparity of ±0.075°. All other features of the RDSs were as previously described.

### Alternating-correlation random dot stereograms

Instead of manipulating binocular correlation over space, as in the half-matched stereogram, we manipulated the binocular correlation over time (Fig 9). The stimulus was presented at a constant refresh rate– 120 Hz—meaning that a new frame of the dynamic RDS was generated every 8.33 ms. The key manipulation was how often the binocular correlation of the dynamic RDS flipped. This could either be at 60, 30, 15, 7.5 or 3.75 Hz. At 60 Hz, a correlation alternation cycle is completed after two frames, at 30 Hz after four frames and so on. Whether a trial started with a correlated or an anticorrelated frame was randomized. For the alternating-correlation RDSs, the surround had the same correlation as the disparity-defined region. We used a dot density of 200% and six disparities: ±0.2275°, ±0.1365°, and ±0.0455°. The stimulus was presented for 500 ms (60 frames) and following the presentation, the observers were asked to report whether the central disk appeared near or far relative to the background using a mouse press.

### Psychophysics experimental procedure

The stimuli were generated in Matlab and displayed using Psychtoolbox [47]. The stimuli were displayed on a 19” Dell Trinitron CRT monitor. For the dot size experiment, the refresh rate of the monitor was 85 Hz and the resolution was 1024 × 768 pixels. The monitor’s luminance output was linearized prior to the experiment. For all experiments, the Michelson contrast was >99%. For the alternating experiment, the refresh rate of the monitor was 120 Hz and the resolution was 800 × 600. Dichoptic presentation was ensured through the use of a simple four-mirror haploscope. In both experiments, the observers indicated using a mouse button press whether the central disk appeared near or far relative to the background. For the statistical testing of the effect of dot size (Fig 7) we used a Monte-Carlo method equivalent to 1-way ANOVA (which does not apply here since the data are binomial proportions). For each observer and dot size we generated random draws from a binomial distribution with a fixed probability, equal to the mean across dot sizes for that observer. We then measured the variance in proportion correct across dot size, generating a distribution of values compatible with the null hypothesis.

### Observers

Four observers participated in the dot size experiment, three of whom were male. Six observers participated in the alternating experiment, four of whom were male. In the alternation experiment, two observers’ data were discarded as they were unable to reliably report depth in 100% correlated stereograms. For both experiments, one of the observers was an author, the rest were naive to the purpose of the experiment. All observers had normal or corrected-to-normal vision using spectacles or contact lenses. Both experiments were approved by the Faculty of Medical Sciences ethics committee at Newcastle University.

### Binocular energy model units

The energy model has been described in detail elsewhere [6,9,13]. Briefly, we modeled the receptive fields of monocular subunits as two-dimensional Gabors with vertical orientation tuning,

$$\rho_{s}\left(x,y\right)=\text{exp}\left(-\frac{{\left(x-x_{0}\pm \frac{\Delta x}{2}\right)}^{2}}{2\sigma_{x}^{2}}-\frac{{\left(y-y_{0}\right)}^{2}}{2\sigma_{y}^{2}}\right)\text{cos}\left(2\pi f\left(x-x_{0}\pm \frac{\Delta x}{2}\right)+\;\varphi \right)$$ (2)

*x*_0_ and *y*_0_ denote the horizontal and vertical receptive field centers, respectively, Δx denotes horizontal disparity and φ denotes phase. *σ*_*x*_ and *σ*_*y*_ denote the horizontal and vertical extent of the receptive fields, respectively, and *f* is the frequency of the Gabor. For all simulations carried out here we used σ_*x*_ = σ_*y*_, and the receptive field centers *x*_0_ and *y*_0_ were placed randomly within the disparity-defined region of the stimulus. No phase disparity was used in any of the models. For Figs 3, 4 and 6 there was no temporal component of the receptive field. For Figs 4b, 6, 7 and 9 we incorporated time by giving each monocular subunit a biphasic temporal kernel as described by Qian and Freeman (2009) [48]

$$\rho_{t}\left(t\right)=\;\{\begin{array}{c} \frac{1}{\Gamma \left(\alpha \right)\tau^{\alpha }}t^{\alpha -1}\text{exp}\left(-\frac{t}{\tau }\right)\text{cos}\left(\omega t+\phi \right)\;\text{if}\;t\geq 0\\ 0\;otherwise \end{array}.$$ (3)

For all simulations where a temporal component was incorporated, we used α = 2.5, *ω* = 4 × 2*π*, ϕ = − π, and τ = 0.035, which gives a temporal kernel with peak response at approximately 4.3 Hz. We chose a biphasic kernel because most V1 neurons have temporal kernels that are bandpass [49]. In Fig 9, we also used the monophasic temporal kernel from Doi et al [15]. The spatial receptive field and temporal kernel were separable giving

$$\rho \left(x,y,t\right)=\rho_{s}\left(x,y\right)\rho_{t}\left(t\right).$$ (4)

Two binocular simple cells were constructed by squaring the sum of two monocular inputs. This produces a binocular simple cell response

$$S={\left(V_{L}+V_{R}\right)}^{2}=V_{L}^{2}+V_{R}^{2}+2V_{L}V_{R}$$ (5)

where *V*_*L*_ and *V*_*R*_ denote the left and right monocular responses, respectively. The disparity tuning of the model arises from the cross-term 2*V*_*L*_*V*_*R*_. The BEM models a complex cell by combining simple cells whose receptive fields are $\frac{\pi }{2}$ out of phase, generating response invariance to stimulus phase.

Combining the simple cell responses, we now have *C* = *S*_1_ + *S*_2_. In order to obtain a cell with an amplitude ratio < 1, we added a static squaring output nonlinearity so that our final model is simply *C*^2^. We computed the disparity tuning curve in Fig 3 by calculating the mean response of the model to 20 000 images displayed at 21 disparities, spanning the range of disparities covered by the neurons’ responses. For generating Fig 4a, we computed the correlated and half-matched response of 30 cells, whose RF size, parameterized by σ, was in the range [0.01°, 0.3°]. We used 11 dot density values, logarithmically spaced from 0.01 to 5.12. The dot size was fixed at 0.09°. We computed the response of each of the 30 cells to 20 000 RDSs per density. For Fig 4b, we computed the correlated and half-matched responses for each cell to dynamic RDSs of 11 different frequencies, ranging from 1Hz to 100Hz. The RF sizes and dot size were the same as for Fig 4a. We obtained the model responses by averaging across 5000 trials per frequency-relative RF size combination, where each trial had a duration of 10s.

### Perceptual decision model

Our model population consisted of 160 neurons tuned to four disparities (±0.48°, ±0.03°). Using only a single neuron at each disparity produced significantly poorer performance than human observers, because performance is limited by the fluctuations in image context from trial to trial. With 40 neurons in each group, and fitted noise levels, performance was comparable to humans. Disparity selectivity was introduced with a position disparity between left and right eyes, with a phase disparity of 0. We made the assumption that receptive field size, parameterized by the standard deviation of the Gaussian envelope σ, scaled with disparity magnitude. Specifically, we had σ = 0.023 + 0.41|Δx|, where |Δx| is the absolute value of the cell’s preferred disparity, measured in degrees. This is similar to previous modeling work that incorporate disparity-size correlations [7]. The specific parameters we have chosen here are not critical—many different sizes and disparities will yield very similar results for the half-matched stimuli, though the exact shape of the psychometric function varies. The frequency of the monocular Gabors scaled inversely with the receptive field size: *f* = 0.3125/σ, in agreement with physiological estimates [46]. The resulting disparity tuning curves, shown in Fig 5, were obtained from computing the average response of each cell to 5000 correlated RDSs per disparity. For the tuning curves, we used disparities 51 disparities spaced from −1° to 1°, with disparities near the peak of the fine tuning curves being sampled more finely. All other stimulus parameters were as previously described.

Gaussian noise was included in the model, where the variance of the noise at any moment in time was proportional to the response of the cell at that time. The response of the *i*th neuron to the *k*th time point is given as

$$P_{ik}=\frac{C_{ik}^{2}}{\langle {\bar{C}}_{i}^{2}\rangle }+\kappa \epsilon_{ik}.$$ (6)

where $C_{ik}^{2}$ is the squared energy model response of the *i*th neuron to the *k*th time point, and $\langle {\bar{C}}_{i}^{2}\rangle$ is the mean response of the squared energy model cell to correlated RDSs at the preferred disparity of the cell, presented at 21.25 Hz. Dividing by the constant scaling factor $\langle {\bar{C}}_{i}^{2}\rangle$ ensures that all cells, have the same maximum response to correlated stimuli at their preferred disparity. In other words, a value of 0.5 in this scheme means that the response was half the mean response to correlated RDSs at the cell’s preferred disparity. *ε*_*ik*_ ∼ *N*(*μ*, *σ*^2^) is the noise in the model, with *μ* = 0 and $\sigma^{2}=C_{ik}^{2}/\langle {\bar{C}}_{i}^{2}\rangle$ (i.e. the variance is proportional to the response magnitude of the cell at any given time). *κ* is a free parameter which governs the magnitude of the noise. This scaling reveals a subtle difference in disparity selectivity with RF size. Because of the final squaring, differences in variability of C lead to differences in mean response. For correlated RDS at the preferred disparity, these fluctuations are correlated in the monocular responses, whereas for uncorrelated RDS they are not. As a result the variability in C is greater for correlated stimuli than for uncorrelated stimuli, and this variability is greater for small RFs than larger ones. Consequently, when scaled by the response to the preferred disparity, smaller RFs show slightly weaker responses to uncorrelated stimuli. Note that the half-matched stimulus introduces additional variation in the binocular correlation, causing responses that are greater than those to uncorrelated dots.

Each neuron also has an antineuron whose response is denoted by *N*_*ik*_. The antineuron response is defined exactly the same as *P*_*ik*_ above, with the same retinal position, except its disparity is the opposite sign. That is to say, if a neuron *P* has a disparity preference of 0.03°, then its antineuron *N* would have a disparity preference of -0.03°. We created an opponent cell by taking the difference of a neuron and its antineuron. To make the decision, each opponent cell’s response was summed across time points

$$R^{\left(i\right)}=\;\Sigma_{k}(P_{ik}-N_{ik}).$$ (7)

*R*^(*i*)^ thus reflects the overall opponent (squared) energy for the *i*th neuron-antineuron pair on a particular trial. If this value is negative, the pair has a mean signal indicative of a negative disparity, and vice versa for positive values.

Within each group of 40 neurons tuned to the same disparity, the models were given non-overlapping RF locations, so that they sampled independent regions of the image. In Fig 6, we used 1.5s trials and summed the responses over time in each model neuron. To obtain the decision, we used a straightforward linear readout of the population response: if the summed activity across the neuron-antineuron pairs was negative, then the decision model would report that the stimulus disparity was negative, and vice versa for positive values:

Ψ={1 if ΣiR(i)>0,0 otherwise. (8)

*R*^(*i*)^ is as defined in Eq 7. Ψ = 0 and Ψ = 1 indicate near and far responses, respectively.

The biphasic temporal kernel employed here had a peak response to 4.3 Hz, meaning that the on-phase of the kernel has a duration of approximately 125 ms. For reference, at 21.25 Hz, the dot pattern is updated every 47.06 ms and at 120 Hz every 8.33 ms. We computed the response of the model to a constant number of RDSs consisting of mixed correlated and anticorrelated dots. Correlation varied from -1 (completely anticorrelated) to +1 (completely correlated) in steps of 0.2. A correlation of 0 in this scheme corresponds to half-matched RDSs. We presented 10 000 trials at each disparity-correlation combination; all other stimulus parameters were as previously described.

## References

[1] Marr D, Poggio T. Cooperative computation of stereo disparity. Science. 1976;194: 283–287. 968482 10.1126/science.968482

[2] Marr D, Poggio T. A computational theory of human stereo vision. Proc R Soc Lond B Biol Sci. 1979;204: 301–328. 37518 10.1098/rspb.1979.0029

[3] Julesz B. Foundation of Cyclopean Perception. 1971.

[4] Parker AJ. Binocular depth perception and the cerebral cortex. Nat Rev Neurosci. 2007;8: 379–391. 17453018 10.1038/nrn2131

[5] Cumming BG, DeAngelis GC. The physiology of stereopsis. Annu Rev Neurosci. 2001;24: 203–238. 11283310 10.1146/annurev.neuro.24.1.203

[6] Qian N, Zhu Y. Physiological computation of binocular disparity. Vision Res. 1997;37: 1811–1827. 9274767 10.1016/s0042-6989(96)00331-8

[7] Allenmark F, Read JCA. Spatial stereoresolution for depth corrugations may be set in primary visual cortex. PLoS Comput Biol. 2011;7: e1002142 10.1371/journal.pcbi.1002142 21876667 PMC3158043

[8] Filippini HR, Banks MS. Limits of stereopsis explained by local cross-correlation. J Vis. 2009;9: 8.1–18. 10.1167/9.1.8 PMC2940423 19271878

[9] Ohzawa I, DeAngelis GC, Freeman RD. Stereoscopic depth discrimination in the visual cortex: neurons ideally suited as disparity detectors. Science. 1990;249: 1037–1041. 2396096 10.1126/science.2396096

[10] Cumming BG, Parker AJ. Responses of primary visual cortical neurons to binocular disparity without depth perception. Nature. 1997;389: 280–283. 9305841 10.1038/38487

[11] Nieder A, Wagner H. Encoding of both vertical and horizontal disparity in random-dot stereograms by Wulst neurons of awake barn owls. Vis Neurosci. 2001;18: 541–547. 11829300 10.1017/s095252380118404x

[12] Haefner RM, Cumming BG. Adaptation to natural binocular disparities in primate V1 explained by a generalized energy model. Neuron. 2008;57: 147–158. 10.1016/j.neuron.2007.10.042 18184571 PMC2344156

[13] Read JCA, Parker AJ, Cumming BG. A simple model accounts for the response of disparity-tuned V1 neurons to anticorrelated images. Vis Neurosci. 2002;19: 735–753. 12688669 10.1017/s0952523802196052

[14] Doi T, Tanabe S, Fujita I. Matching and correlation computations in stereoscopic depth perception. J Vis. 2011;11: 1. 10.1167/11.3.1 21367941

[15] Doi T, Takano M, Fujita I. Temporal channels and disparity representations in stereoscopic depth perception. J Vis. 2013;13: 26. 10.1167/13.13.26 24281242

[16] Doi T, Fujita I. Cross-matching: a modified cross-correlation underlying threshold energy model and match-based depth perception. Front Comput Neurosci. 2014;8: 127 10.3389/fncom.2014.00127 25360107 PMC4197649

[17] Smallman HS, MacLeod DI. Size-disparity correlation in stereopsis at contrast threshold. J Opt Soc Am A Opt Image Sci Vis. 1994;11: 2169–2183. 7931758 10.1364/josaa.11.002169

[18] Prince SJ, Eagle RA. Size-disparity correlation in human binocular depth perception. Proc Biol Sci. 1999;266: 1361–1365. 10445290 10.1098/rspb.1999.0788 PMC1690069

[19] Tyler CW. Depth perception in disparity gratings. Nature. 1974;251: 140–142. 4420707 10.1038/251140a0

[20] Prince SJD, Cumming BG, Parker AJ. Range and mechanism of encoding of horizontal disparity in macaque V1. J Neurophysiol. 2002;87: 209–221. 11784743 10.1152/jn.00466.2000

[21] Hibbard PB, Scott-Brown KC, Haigh EC, Melanie A. Depth Perception Not Found in Human Observers for Static or Dynamic Anti-Correlated Random Dot Stereograms. PLoS One. 2014;9: e84087 10.1371/journal.pone.0084087 24416195 PMC3885516

[22] Read JC, Eagle RA. Reversed stereo depth and motion direction with anti-correlated stimuli. Vision Res. 2000;40: 3345–3358. 11058733 10.1016/s0042-6989(00)00182-6

[23] Tanabe S, Yasuoka S, Fujita I. Disparity-energy signals in perceived stereoscopic depth. J Vis. 2008;8: 22.1–10. 10.1167/8.3.22 18484828

[24] Cumming BG, Shapiro SE, Parker AJ. Disparity detection in anticorrelated stereograms. Perception. 1998;27: 1367–1377. 10505181 10.1068/p271367

[25] Uka T, DeAngelis GC. Contribution of area MT to stereoscopic depth perception: choice-related response modulations reflect task strategy. Neuron. 2004;42: 297–310. 15091344 10.1016/s0896-6273(04)00186-2

[26] Shadlen MN, Britten KH, Newsome WT, Movshon JA. A computational analysis of the relationship between neuronal and behavioral responses to visual motion. J Neurosci. 1996;16: 1486–1510. 8778300 10.1523/JNEUROSCI.16-04-01486.1996 PMC6578557

[27] Gold JI, Shadlen MN. The Neural Basis of Decision Making. Annu Rev Neurosci. 2007;30: 535–574. 17600525 10.1146/annurev.neuro.29.051605.113038

[28] Read JCA. A Bayesian model of stereopsis depth and motion direction discrimination. Biol Cybern. 2002;86: 117–136. 11911114 10.1007/s004220100280

[29] Goris RLT, Putzeys T, Wagemans J, Wichmann FA. A neural population model for visual pattern detection. Psychol Rev. 2013;120: 472–496. 10.1037/a0033136 23915083

[30] Banks MS, Gepshtein S, Landy MS. Why is spatial stereoresolution so low? J Neurosci. 2004;24: 2077–2089. 14999059 10.1523/JNEUROSCI.3852-02.2004 PMC6730432

[31] Kane D, Guan P, Banks MS. The limits of human stereopsis in space and time. J Neurosci. 2014;34: 1397–1408. 10.1523/JNEUROSCI.1652-13.2014 24453329 PMC3898296

[32] Harris JM, Parker AJ. Independent neural mechanisms for bright and dark information in binocular stereopsis. Nature. 1995;374: 808–811. 7723825 10.1038/374808a0

[33] Read JCA, Vaz XA, Serrano-Pedraza I. Independent mechanisms for bright and dark image features in a stereo correspondence task. J Vis. 2011;11: 4–4. 10.1167/11.12.4 21984818

[34] Nakayama K, Shimojo S. da Vinci stereopsis: depth and subjective occluding contours from unpaired image points. Vision Res. 1990;30: 1811–1825. 2288092 10.1016/0042-6989(90)90161-d

[35] Fricke TR, Siderov J. Stereopsis, stereotests, and their relation to vision screening and clinical practice. Clin Exp Optom. 1997;80: 165–172.

[36] Fawcett SL. An evaluation of the agreement between contour-based circles and random dot-based near stereoacuity tests. J AAPOS. 2005;9: 572–578. 16414526 10.1016/j.jaapos.2005.06.006

[37] Giaschi D, Narasimhan S, Solski A, Harrison E, Wilcox LM. On the typical development of stereopsis: fine and coarse processing. Vision Res. 2013;89: 65–71. 10.1016/j.visres.2013.07.011 23891704

[38] Ogle KN. On the limits of stereoscopic vision. J Exp Psychol. 1952;44: 253–259. 13000066 10.1037/h0057643

[39] Westheimer G, Tanzman IJ. Qualitative depth localization with diplopic images. J Opt Soc Am. 1956;46: 116–117. 13295877 10.1364/josa.46.000116

[40] Read JCA. Stereo vision and strabismus. Eye. 2015;29: 214–224. 10.1038/eye.2014.279 25475234 PMC4330289

[41] Zhang Z-L, Cantor CRL, Schor CM. Perisaccadic stereo depth with zero retinal disparity. Curr Biol. 2010;20: 1176–1181. 10.1016/j.cub.2010.04.060 20619816

[42] van Ee R, Erkelens CJ. Stereo-vision: head-centric coding of retinal signals. Curr Biol. 2010;20: R567–8. 10.1016/j.cub.2010.05.001 20619811

[43] Doi T, Tanabe S, Fujita I. Reversed depth perception in anticorrelated random-dot stereograms: when it is perceived and when it is not. 2014; Available: http://www.plosone.org/annotation/listThread.action?root=78048

[44] Janssen P, Vogels R, Liu Y, Orban GA. At least at the level of inferior temporal cortex, the stereo correspondence problem is solved. Neuron. 2003;37: 693–701. 12597865 10.1016/s0896-6273(03)00023-0

[45] Tanabe S, Umeda K, Fujita I. Rejection of false matches for binocular correspondence in macaque visual cortical area V4. J Neurosci. 2004;24: 8170–8180. 15371518 10.1523/JNEUROSCI.5292-03.2004 PMC6729782

[46] Prince SJD, Pointon AD, Cumming BG, Parker AJ. Quantitative analysis of the responses of V1 neurons to horizontal disparity in dynamic random-dot stereograms. J Neurophysiol. 2002;87: 191–208. 11784742 10.1152/jn.00465.2000

[47] Kleiner M, Brainard D, Pelli D, Ingling A, Murray R, Broussard C. What’s new in Psychtoolbox-3. In: Google Code [Internet]. 2007. Available: https://psychtoolbox-3.googlecode.com/svn-history/r1555/beta/Psychtoolbox/PsychDocumentation/Psychtoolbox3-Slides.pdf

[48] Qian N, Freeman RD. Pulfrich phenomena are coded effectively by a joint motion-disparity process. J Vis. 2009;9: 24.1–16. 10.1167/9.5.24 PMC2941507 19757902

[49] Hawken MJ, Shapley RM, Grosof DH. Temporal-frequency selectivity in monkey visual cortex. Vis Neurosci. 1996;13: 477–492. 8782375 10.1017/s0952523800008154

